# The Effects of Choice on the Reading Comprehension and Enjoyment of Children with Severe Inattention and no Attentional Difficulties

**DOI:** 10.1007/s10802-021-00835-8

**Published:** 2021-06-21

**Authors:** Myrofora Kakoulidou, Frances Le Cornu Knight, Roberto Filippi, Jane Hurry

**Affiliations:** 1 Department of Psychology and Human Development, Institute of Education, UCL, 25, Woburn Square, London, WC1H 0AA UK; 2 Centre for Psychological Approaches for Studying Education, School of Education, University of Bristol, 35, Berkeley Square, Bristol, BS8 1JA UK

**Keywords:** Attention Deficit Hyperactivity Disorder (ADHD), Inattention, Reading comprehension, Reading motivation, Choice, Situational interest

## Abstract

It has been proposed that enhancing motivation supports the learning of children with Attention Deficit Hyperactivity Disorder (ADHD). Less is known if inattentive children with no ADHD diagnosis may similarly benefit, when being motivated to engage in an academic task. Using a repeated-measures design, this study investigated the effects of text choice as an intrinsic motivator on the reading comprehension and enjoyment of Year 4 children attending mainstream primary schools (*N* = 92; aged 8–9 years; 48 boys); comparing those with no attentional difficulties and severe inattention. We hypothesized that 1) choice would increase reading comprehension and enjoyment 2) choice would increase the reading comprehension and enjoyment of children both with severe inattention and no attentional difficulties 3) choice effects would be significantly greater for children with severe inattention than those with no attentional difficulties. Children participated in a reading intervention that included a Choice (experimental) and a No Choice (control) condition. Child inattention was measured via a Virtual Reality Continuous Performance Task (Omission errors, Reaction Time Variability) and Teacher Ratings. Choice significantly increased reading comprehension, but not enjoyment compared with no choice. Choice improved the reading comprehension of children with both severe inattention and no attentional difficulties. Choice did not benefit the reading of severely inattentive children more than that of children with no attentional difficulties. These findings underline the educational benefits of choice for young readers both with severe inattention and no attentional difficulties, which are further discussed drawing on existing theory and research.

**Supplementary Information:**

The online version contains supplementary material available at 10.1007/s10802-021-00835-8.

Attention Deficit Hyperactivity Disorder (ADHD) is one of the most common childhood neurodevelopmental conditions characterized by severe degrees of inattention and/or hyperactivity/impulsivity that are inconsistent to the child’s developmental level (American Psychiatric Association [APA], 2013). ADHD affects approximately 5–7% of young people worldwide (Willcutt, 2012). According to parent reports, the estimated prevalence of children diagnosed with ADHD in the UK is at 1.5% (Russell et al., 2013). Children with ADHD are at higher risk of reading underachievement than their neurotypical peers (Miller et al., 2013) as reading involves a number of attentional mechanisms, which require one’s ability to focus on relevant textual information (selective attention) irrespective of any distractors (behavioral inhibition) and maintain attention over time (sustained attention) (Kendeou et al., 2014).

Reading disabilities (RD) and ADHD co-occur in both community and clinical samples (Sexton et al., 2012). This relationship offers a further critical insight into those factors that may affect reading. Readers with co-occurring ADHD and RD struggle primarily with slow processing speed (Willcutt et al., 2010). Readers with RD alone present typically difficulties with phonological processing (Purvis & Tannock, 2000), although other factors such as verbal reasoning, naming speed, processing speed and working memory may exacerbate their reading difficulties (Willcutt et al., 2010). Readers with ADHD, without co-occurring RD, do not commonly experience phonological difficulties (Purvis & Tannock, 2000). These readers usually struggle when they read long texts (Cherkes-Julkowski et al., 1995), achieve lower, within the average, rates on measures of accuracy and silent reading comprehension (Ghelani et al., 2004) as well as experience difficulties in the recall of central ideas (Miller et al., 2013). Young readers with ADHD may invest more cognitive processes in sustaining attention, and therefore they are left with limited resources for reading comprehension.

Whilst research focus has been traditionally on the reading of children with diagnosed ADHD, children who display early inattention and hyperactivity/impulsivity between ages 5 to 7, but do not hold a diagnosis, may later struggle with their literacy and reading (Hurry et al., 2018; Merrell et al., 2017). Studies with children attending mainstream schools, aged 6 to 10 years old, reported both the direct association of attention with text-level reading comprehension and the indirect association of attention with reading via oral language comprehension (Kieffer et al., 2013) and word reading growth (Miller et al., 2014), even when other reading-related factors such as phonological awareness, vocabulary and hyperactivity were considered. Inattention may serve as a greater predictor of reading than hyperactivity/impulsivity, even after controlling for intellectual ability, prior reading achievement and parental involvement (Lahey & Willcutt, 2010; Rabiner & Coie, 2000; Sims & Lonigan, 2013). In the absence of a diagnosis, the reading difficulties of inattentive children could go unnoticed in classroom, as their inattentive behavior is less expected to overtly interfere with teacher instruction (Merrell et al., 2017). Thus, although at risk of reading underachievement, these children may not receive extra reading support in classroom.

Medication (e.g. methylphenidate) and behavioral interventions (e.g. parent training and positive reinforcement) are designed to reduce diagnosed inattention and/or hyperactivity/impulsivity, rather than target specifically learning (The MTA Cooperative Group, 2004). School-based academic interventions that address directly learning, such as task/ instructional modification and peer tutoring may be more educationally appropriate for inattentive children with no ADHD diagnosis (Dupaul et al., 2012). These children are less likely to be medicated or receive behavioral interventions that traditionally aim to address more challenging behaviors observed in some children with diagnosed ADHD.^1^

Drawing on research with children in mainstream schools, academic interventions that promote motivation could support inattentive children with their learning. In the context of reading, *motivation* is a multifaceted construct and describes those factors that drive a child to engage in reading (Wigfield et al., 2016). Motivated children have a greater likelihood to become proficient readers who read for pleasure (Department for Education [DfE], 2012). Key theories such as the Delay aversion theory and the Optimal stimulation theory posit that ADHD has a motivational component (Sonuga-Barke, 2005; Zentall, 1975), with young people with ADHD being at greater risk of poor academic motivation than their neurotypical peers (Smith & Langberg, 2018). Lack of motivation in ADHD is probably due to a lower than average activity in the brain’s reward circuitry mechanisms, specifically in the dopaminergic pathway (Volkow et al., 2011).

According to the Delay aversion theory, people with ADHD engage in inattentive and hyperactive/impulsive behaviors to reduce negative feelings associated with delay, commonly expressed though their choice of immediate non-salient over delayed salient rewards (Marx et al., 2018). In contexts where avoiding or escaping delay is not possible, they may seek stimulation to gain immediate gratification, either through paying attention to the interesting aspects of their environment or adapting this to make it interesting (Sonuga-Barke, 2005). Delay aversion motivates stimulation seeking. The Optimal stimulation theory similarly emphasises the stimulation-seeking aspect of ADHD and posits that people with ADHD are in higher need of stimulation to reach an optimal level of arousal to sustain attention during an activity than the neurotypical ones (Zentall, 1975). Decreased dopamine activity (Volkow et al., 2011) motivates them to seek arousal/stimulation. When stimulation is not provided, they would engage in inattentive and/or hyperactive/impulsive behaviors to meet this stimulation threshold (Beike & Zentall, 2012). Difficulty in maintaining attention in classroom could thus stem to a degree from lack of sufficient environmental stimulation. Regarding reading, poor motivation may provide an explanation for the reading difficulties of children with severe attentional difficulties and/or ADHD, and thus external sources of stimulation and motivation may be needed to secure optimal reading experiences and performance.

Motivational interventions to date have found that extrinsic motivators such as rewards and feedback increase motivation and cognitive performance (e.g. accuracy) in behavioral tasks (e.g. Go/No-Go tasks, Continuous Performance Tests) for both neurotypical children and children with ADHD, with these effects being more salient for the latter group (Marx et al., 2018). In a literature review of 22 studies with 1181 children aged 5 to 14 years old, Luman et al. (2005) found that external reinforcers (e.g. rewards) affected the performance of neurotypical children less than children with ADHD, with the former group performing well irrespective of the presence of external reinforcers. These findings encourage further exploring the effects of different motivational sources (e.g. intrinsic motivators) in the learning of non-diagnosed inattentive children. Reading practices that enhance motivation could provide an alternative way for educators to improve the reading of inattentive children.

*Choice* has long been regarded as a central motivational instructional practice that encourages children to exercise a sense of ownership over what to read, how to read or when to read (Flowerday et al., 2004; Wigfield & Guthrie, 1997). Children who have a sense of ownership during learning show greater affective (e.g. enjoyment, effort) and cognitive (e.g. task performance) engagement (Ryan & Deci, 2000). The role of choice during learning has been explored extensively within the influential *Four-Phase Model of Interest Development* (Hidi & Renninger, 2006), which posits that choice is a trigger of situational interest. *Situational interest* describes the first phase of personal interest development, which can be generated by contextual features such as choice and may or may not last (Hidi & Renninger, 2006). Instructional practices that promote continuously situational interest including choice have been shown to increase intrinsic motivation (one’s intrinsic need to engage in a task for its own sake out of personal interest), willingness to re-engage with a task, cognitive performance and overall reading (Renninger & Hidi, 2016). Theory and research with neurotypical children underline the positive choice effects on affective and cognitive engagement. Fridkin (2018) explored the effects of text choice on the reading comprehension (assessed via comprehension questions) and enjoyment (assessed via a self-report) of 110 children aged 8 to 9 years old. During a repeated measures design that controlled for prior story interest and story knowledge, children participated in a Choice (experimental) condition, where they chose between two stories and a No Choice (control) condition, where a story was pre-assigned by the researcher. Children achieved greater reading comprehension and enjoyment in the Choice than the No Choice condition. Choice effects on reading comprehension and enjoyment were not moderated by baseline reading ability and sex.

Interpreting findings in research, however, is complex as there are certain conditions under which choice could support or undermine potential academic outcomes. For example, in order for choice to increase learning, this should represent a meaningful act of choosing that is to involve some degree of considered thinking and critical evaluation of the alternative options (Katz & Assor, 2007). Therefore, when examining choice effects, factors such as prior story interest and story knowledge should also be considered. Despite these methodological limitations, choice could serve as an effective practice to build interest (and subsequent motivation) for tasks that are not genuinely interesting for a learner (Patall, 2013). Thus, choice may represent a promising avenue of educational intervention for children at risk of poor motivation and reading underachievement such as inattentive children.

Literature in ADHD provides some evidence for the educational importance of motivational and autonomy-supportive practices (e.g. teacher avoids controlling language and the child is free to express ideas) including choice. In a correlational study with 302 adolescents with and without diagnosed ADHD (*M* age = 13.20), Smith et al. (2019) found that higher self-reported intrinsic motivation for knowledge in children with ADHD was significantly associated with higher reading accuracy. For adolescents without ADHD, lower self-reported intrinsic motivation for accomplishment was associated with lower reading accuracy. In both groups, lack of motivation (amotivation) was associated with lower parent-reported homework performance. Despite the fact that this study was correlational and not experimental, it underlines the beneficial role of intrinsic motivators such as choice for the reading of children with and without diagnosed ADHD. Using a community sample of 117 children aged 6 to 11 years old, Rogers and Tannock (2018) found that children with severe non-diagnosed inattention and hyperactivity/impulsivity as rated by teachers perceived their classrooms as providing limited opportunities for autonomy compared with peers with mild such characteristics, after controlling for age, co-occurring conduct problems and reading ability. Autonomy-supportive instructional practices provide opportunities for meaningful choice, avoid controlling language, validate children’s perspectives (Sierens et al., 2009) and have been associated with increased academic outcomes in neurotypical children (see Su & Reeve, 2011 for a meta-analysis). Therefore, in the study of Rogers and Tannock (2018), negative perceptions of the school environment could undermine child’s learning and create a vicious circle of school failures. Studies in ADHD underline the positive effects of choice on task-related behavior (e.g. on-task behavior) and advocate for the potential benefits of choice for learning (Dunlap et al., 1994; Powell & Nelson, 1997; Shogren et al., 2004). However, the great majority of these choice studies are dated and have employed single case study designs. These case studies have also tested choice effects with children with ADHD who are more likely to experience challenging behaviors; nevertheless, predominantly inattentive children are less likely to present such behaviors. Research around choice effects on the learning and particularly reading of non-diagnosed inattentive children has been neglected. Therefore, it remains to be seen whether choice could also benefit the reading of these children. Lack of evidence-based educational reading interventions with non-diagnosed inattentive children stresses the need to explore further this significant learning area. Despite the complexity of the area, the idea that interest can develop and that instructional practices such as choice can play an important role for its development could have important implications in reading research.

To our knowledge, this study is the first to explore choice effects on the reading comprehension and enjoyment of children with attentional difficulties in mainstream schools. This study replicated and extended Fridkin’s study (2018), which found significant effects of choice on the reading comprehension and enjoyment of eight-to-nine year old children in mainstream school. The present study addressed three aims. The first aim was to investigate choice effects on the reading comprehension and enjoyment, replicating Fridkin’s findings (2018). *Hypothesis 1* is that choice will increase children’s reading comprehension and enjoyment. The second aim was to examine choice effects on the reading comprehension and enjoyment of those children with attentional difficulties. Whilst a relationship between inattention and reading in children in mainstream schools has been observed (Rabiner et al., 2016), our knowledge is limited as to what practices can support the reading of inattentive children. *Hypothesis 2* is that choice will improve the reading comprehension and enjoyment of children with both severe inattention and no attentional difficulties. The third aim was to test for any differences in choice effects on the reading comprehension and enjoyment of children in mainstream schools with severe inattention compared with those with no attentional difficulties. Theory predicts and there is some evidence to support that children with ADHD benefit more from motivation (particularly extrinsic motivation including rewards) than their neurotypical peers (Luman et al., 2005). Little is known, however, if this also applies to severely inattentive children, who do not hold a diagnosis. *Hypothesis 3* is that severely inattentive children will benefit more from choice than children with no attentional difficulties.

## Method

### Participants

Using the G* Power 3.1 software, an a priori analysis showed that a minimum sample size of 42 children was necessary to report medium effects (${\eta }_{p}^{2}$= 0.06) and significant interactions between Choice and Attention variables at a power (alpha) of 0.05 (Faul et al., 2007). Children were recruited from six classrooms of two mainstream mixed primary community schools in London rated as ‘Good’ based on the most recent Ofsted report. The final sample size consisted of 92 Year 4 children (*M* age = 8.86 years; *SD* = 0.34; 48 boys). Year 4 children were sought as reading motivation and enjoyment are more likely to decline towards the end of primary phase (Sainsbury & Schagen, 2004), and thus these children may benefit more from motivational practices such as choice. Further to this, most studies explored choice effects on academic motivation and performance with middle school, college students and adults (Flowerday & Schraw, 2003; Flowerday et al., 2004; Patall, 2013). Here, we investigated whether choice would benefit similarly the learning of primary-aged children (Fridkin, 2018). One-hundred nineteen children returned parental opt-in consent. Due to the focus of the study on a community sample with severe and no attentional difficulties, the sample was finalised after excluding 11 children (6 boys, 5 girls, 10.19%) who received an Education, Health and Care plan (EHC) or a Special Education Needs (SEN) support. Sixteen children were further excluded as teacher data (*N* = 5), data on the virtual reality task (*N* = 6) and reading data (*N* = 5) could not be collected due to child absence or technical difficulties. Ninety-two children were finally included in the study. Additional information about the participant characteristics was provided by teachers. The majority of children were of Black ethnic background (*N* = 35, 38.04%), others had a White ethnic background (*N* = 21; 22.83%), others an Asian ethnic background (*N* = 19; 20.65%), others a Mixed ethnic background (*N* = 9; 9.8%) and others had any other ethnic background (*N* = 6; 6.5%). Of the 92 participating children, 12 children (13%) were eligible to free school meals, 16 children were on pupil premium (17.4%) and 49 children (53.3%) had English as an Additional Language (EAL).

## Measures

### The New Group Reading Test (NGRT)

 This 48-item standardised reading test includes a 20-item sentence completion section and a three- passage comprehension section, both in a multiple choice format to control for writing ability (Burge et al., 2010). The maximum score is 48. Raw scores were converted to standardised scores by age (*M* = 100, *SD* = 15). Cronbach’s alpha was previously reported to be above 0.9 for both test sections ensuring high reliability (GL Assessment, 2018). In this study, Cronbach’s alpha gave a value of 0.90 ensuring the effectiveness of this measure to assess reading comprehension. Validity can be demonstrated based on the high internal consistency and test–retest correlations reported previously (*r* = 0.85, GL Assessment, 2018).

### Adapted Version of the Motivation for Reading Questionnaire (MRQ)

 An adapted version of the widely used MRQ measured aspects of baseline reading motivation such as reading efficacy, curiosity and involvement with reading (Wigfield & Guthrie, 1997). This version included 38 items, excluding items that explored solely extrinsic motivation, with two practice questions. The response format ranged from 1 = *very different from me* to 4 = *a lot like me* and scores were computed for the motivational scale by using the sum of the items. Six items made negative statements related with reading and thus they were reverse coded. In a series of studies with primary-aged children, reliability for the original version ranged between 0.43 and 0.81 (Guthrie et al., 2007; Wigfield & Guthrie, 1997). Construct validity was also reported (Wigfield & Guthrie, 1997) and the majority of correlations between the reading motivation aspects were positive and varied from low to moderately strong. We used the Expectation–Maximisation (EM) method to treat MRQ data that were not completed at random (Scheffer, 2002) and avoid any unnecessary data loss. Complete MRQ data after imputation were finally used. Cronbach’s alpha gave a value of 0.84 indicating good-to-excellent reliability. Concurrent validity was assessed by calculating the correlation between the MRQ and the Enjoyment questionnaire applied in the No Choice (control) condition. A moderate correlation was found between the MRQ and the Enjoyment questionnaire, *r* = 0.51 (Cohen, 1988).

### AULA: Advanced Virtual Reality Tool for the Assessment of Attention

AULA was administered to secure objective assessments of children’s ability to sustain cognitive performance over time (sustained attention) in the presence of realistic classroom distractors and not for clinical purposes (see Iriarte et al., 2016 for a detailed description of the measure). AULA is an ecologically valid measure that has proved effective at evaluating inattention and identifying differences between neurotypical children and children with ADHD (Areces et al., 2018). Each child wears a set of 3-D virtual glasses with movement sensors and holds a response button. AULA uses a standard Continuous Performance Task (CPT) paradigm and comprises: a) a No-X paradigm, where a response is required on 80% trials, in this case if they do not see or hear ‘apple’ and b) an X paradigm, where a response is required on 20% trials, in this case if they see or hear ‘seven’. Stimuli are presented in the visual and auditory domains at equal frequencies, and randomised everyday visual and auditory distractors (e.g. classmates talking, teacher walking in the room) occur on 50% of trials. AULA generates a number of variables. For the purposes of this study, *Omission errors* and *Reaction Time Variability (RTV)* were selected as we were interested in the attentional difficulties specifically, rather than hyperactivity/impulsivity. *Omission errors* occur when the child fails to respond on trials, which require a response. These are indicative of poor sustained attention. *RTV* has been associated with ADHD, poor word reading, reading fluency and reading comprehension in children with ADHD (Tamm et al., 2014) and reflects occasional lapses, such that greater RTV represents more attentional lapses. These momentary lapses in sustained attention are not severe enough to produce omission errors, and so are captured as longer response times (RTs). Using discriminant analysis, Areces et al. (2018) found that 76.1% of children were correctly classified (66% from the control group and 89.5% from the ADHD group) based on Omission errors. Convergence with the CPT of Conners is also significant (*p* < 0.01 and *p* < 0.05), *r* ranging between 0.6 and 0.8 in a mixed sample of 57 children with ADHD (Diaz-Orueta et al., 2014). In the Iriarte et al. (2016) study with a community sample of 141 9-year-old children, descriptive statistics for Omission errors showed *M* = 23.51, *SD* = 24.23 and for RTV, *M* = 376.42 and *SD* = 75.59.

### Conners 3rd Edition-Teacher (Conners 3-T) Rating Scale (Short Version)

Teachers completed the short version of the Conners 3 Teacher Rating Scale (Conners, 2008) that provides information about children’s inattention and hyperactivity/impulsivity based on the DSM-V (American Psychiatric Association, 2013). Teachers’ accounts were the most informative for this study that explored whether motivation could enhance the reading outcomes of inattentive children in classroom. The inattention scale was selected. This included 5 items scored on a 4-point Likert-type scale that ranged from 0 = *not at all* to 3 = *very much true*. Higher scores showed greater inattention. Previously, Cronbach’s alpha gave values ranging from 0.84 to 0.94 for the Teacher scale (short version) showing high reliability (Izzo et al., 2019). In this study, Cronbach’s alpha gave a value of 0.96 for the whole scale and a value of 0.91 for the Teacher Inattention scale. Raw scores were converted to T scores based on age and sex and used in analyses. T scores have a mean of 50 and a standard deviation of 10. T scores equal and/or above 65 indicate elevated attentional difficulties (more concerns than are typically reported).

### Stories, Reading Comprehension Questions and Scoring

Two short stories written by Fridkin (2018), matched for word length and difficulty level and of similar structure were administered to assess reading comprehension. Story A (Wishing on a Star/A Snowy Adventure) included 682 words and 7 pictures, while Story B (Just Another Ordinary Day/Something’s going on) comprised 627 words and 7 pictures. Two different titles, cover pages, prologues and short story reviews (these gave generic positive reviews about the stories and were developed based on children’s comments in Fridkin’s pilot study [2018]) were created for each story to be administered in the Choice condition. Following adjustments to Fridkin (2018) comprehension questions, a set of 16 multiple choice and open-ended questions were developed including a total of 19 items for each story. The maximum score was 19. Internal consistency was 0.77 for Story A and 0.66 for Story B. This level of internal consistency was moderate- to- good considering the small number of items (Koo & Li, 2016). Concurrent validity was explored by measuring the correlation between the two stories and the NGRT (standardised). Pearson correlation between Story A and the NGRT was *r* = 0.66, correlation between Story B and the NGRT was *r* = 0.53 and correlation between Story A and Story B was *r* = 0.46. Statistics showed a medium correlation strength (Cohen, 1988) providing evidence for the concurrent validity of the measure given the small number of items. A possible explanation for the medium-strength correlations is that the NGRT assesses both passage comprehension and sentence completion, whereas the story questions measured only reading comprehension. Difference scores were computed subtracting reading comprehension scores in No choice condition by comprehension scores in Choice condition.

### The ‘Story Enjoyment/Interest’ Scale

Children rated the story they have just read via a 13-item Likert scale, designed based on the questionnaires of Fridkin (2018) and Wigfield and Guthrie (1997). The format ranged from 1 = *very different from me* to 4 = *a lot like me*. Total scores were summed for each child across both conditions. Enjoyment and interest have been considered as two closely related, but distinct motivational constructs (Renninger & Hidi, 2016). *Interest* motivates exploration and novelty seeking, whereas *enjoyment* describes the satisfaction experienced during an activity. Cronbach’s alpha value of 0.79 suggested a good reliability. Concurrent validity was measured by checking the correlation between the 13-item scale across both conditions and the MRQ. The ‘Story Enjoyment/Interest’ scale in the No Choice condition had a moderate correlation with the MRQ, *r* = 0.51 and the same scale in the Choice condition had also a moderate correlation with the MRQ, *r* = 0.50. The moderate correlation observed between the ‘Story Enjoyment/Interest’ scale and the MRQ, may be due to the fact that the former explored only aspects of interest and enjoyment, whereas the latter examined further aspects of motivation as mentioned earlier. Difference scores were computed subtracting enjoyment scores in No Choice condition by enjoyment scores in Choice condition.

## Design

A repeated measures design with Choice as the within-subjects variable and Inattention, measured via Teacher-rated Inattention, Omission errors and RTV, as the between-subjects variable tested for any differences in reading comprehension and reading enjoyment due to choice provision in children with severe inattention and children with no attentional difficulties. Children were randomly assigned to the Choice or No Choice condition. A crossover design was applied and all children experienced both conditions by the end of the intervention. Half the children completed the Choice condition first and the No Choice condition after and the other half of the children vice versa. Half of the children received Story A in the Choice condition, half Story B. All four text versions (two for each story) were rotated and administered across both conditions, counterbalancing the condition order.

## Procedure 

This study received ethical approval by the Research and Ethics Committee at UCL, Institute of Education. Information letters and opt-in consent forms were sent via teachers to parents/carers before the study. The study began in December 2017 and lasted until January 2019. In the first school visit, children completed the NGRT first and then the MRQ. In the second visit, children of each classroom were randomly assigned to the Choice or No Choice condition. In the first classroom, children participated in the experimental condition (Choice) with the control condition (No Choice) following on a different date (third visit). In the second classroom, children took part in the control condition first with the experimental one taking place after (third visit). Children completed all the tasks individually. The reading tasks were administered in children’s regular classroom during morning sessions that lasted approximately 1 h each. The three visits were between two to seven days apart. Further visits took place in-between the three main visits, during which children completed the AULA task individually.

In the Choice (experimental) condition, children received two A4 envelopes. Stapled to the front of the envelope they found a different cover page for each story, a different prologue for each story and two different child reviews. The choice was a perceived choice; the actual stories inside the envelopes were the same; only the titles, prologues and reviews were different. Perceived choice enables to control for background knowledge and individual/topic interest. Children had 5 min to read through the stapled material to ensure more meaningful choice. The number of stories was restricted to two as limitless choice could be burdensome and potentially demotivating (Clark & Phythian-Sence, 2008). Once children made their decision, they opened the envelope containing a full version of the chosen story and a set of questions. Children read the story and answered the questions within 45 min. In the No Choice (control) condition, children received one of the two stories. They had 45 min to complete the reading task. The procedure was identical to that of the experimental condition, except for the story selection. In both conditions, after the completion of the reading tasks, children rated the story they had just read using the ‘Story Enjoyment/Interest’ scale. Children provided verbal consent and completed the AULA individually in a quiet school room during morning or afternoon 20 min sessions. Teacher scales were collected before, during or after the intervention. Children received a certificate of participation and teachers thank-you vouchers.

## Data Analysis

Data^2^ were analysed using the IBM SPSS Statistics 25 package. In the Exploratory Data Analysis (EDA), the Shapiro–Wilk Test showed that data were not always normally distributed (e.g. Omission errors). However, no bimodal or multimodal distributions were observed in histograms (some normal despite the skewness and kurtosis was noted). Based on the Central Limit Theorem (*N* ≥ 30), the assumption of normality is not necessary (Lumley et al., 2002) in moderately large samples (*N* = 92). Thus, parametric tests were employed. Descriptive statistics and bivariate correlations among all study variables were initially explored. In order to address *Hypothesis 1*, we used a repeated measures ANOVA with Choice as the within-subjects variable. For *Hypothesis 2* and *3,* we followed two analytical methods. First, we applied a whole-sample analysis in order to preserve the continuous nature of the Attention variables, by checking the correlations between the difference scores in Reading comprehension and Enjoyment and the Attention measures to test for any relationships between Attention and Choice. Greater difference scores would indicate that children achieved greater Reading comprehension and Enjoyment in Choice than No Choice condition and vice versa. Use of difference scores could help infer an interaction from the correlation; significant positive correlations between the difference scores in Reading comprehension and Attention variables and between the difference scores in Enjoyment and Attention variables would suggest that there is an interaction effect such that severely inattentive children would benefit more from Choice than No Choice than children with no attentional difficulties. This method aligns with dimensional approaches to ADHD (Gathercole et al., 2018).

After this, three mixed 2 × 3 repeated measures ANOVA tests with a within-subjects variable (Choice) and a trichotomized between- subjects variable (No attentional difficulties, Some inattention, Severe inattention) for each of the three attention variables (Teacher-rated Inattention, Omission errors and RTV) were employed to test the three hypotheses. Contrary to the median split method, trichotomization is a more refined method that enables to discretise a continuous variable (inattention as measured by Conners 3 and AULA) into three groups based on percentiles and then compare the upper and lower third group (Hagmar et al., 1994). This method led into three groups labelled as ‘No Attentional difficulties’, ‘Some Inattention’ and ‘Severe Inattention’. All three groups were included in the ANOVA analysis to consider the full sample (Proctor et al., 2010).^3^ Following the theory of trichotomization, multiple comparisons using Bonferroni’s corrections (0.05/3 = 0.017 for the group comparisons) were observed to test the effects of Choice on the Reading comprehension and Enjoyment of the ‘No Attentional difficulties’ group and the ‘Severe Inattention’ group. We employed the trichotomization method to maximize the comparison between the two main groups of interest. The total sample size of the two groups (*N* = 61) still met power calculation requirements as indicated by the power analysis (*N* ≥ 42). Table 1 shows the means and standard deviations for the ‘No Attentional difficulties’ and ‘Severe Inattention’ groups across the Attention variables. The ‘No Attentional difficulties’ group was rated by teachers within the average range based on the Conners 3 thresholds for T scores (40 to 59), while the ‘Severe Inattention’ group showed elevated attentional difficulties (T ≥ 65). Based on the Iriarte et al. (2016) study, mean scores for Omission errors and RTV were reported within the average range for the ‘No Attentional difficulties’ group, whereas these scores were well above the average for the ‘Severe Inattention’ group (more than two *SD* above the mean for Omission errors and more than one *SD* above the mean for RTV). Such congruence between the statistics reported in our study and previous research provides further support for the reliability of our trichotomization method.

**Table 1:** Means and standard deviations for the attention variables across the two groups

Measure	Group	*N*	*M (SD)*
Conners 3 Teacher-rated Inattention	No Inattention Severe Inattention	33 28	43.10(1.01) 65.64(8.37)
AULA Omission errors	No Inattention Severe Inattention	30 31	8.67(3.59) 80.16(19.24)
AULA RTV	No Inattention Severe Inattention	30 31	321.18(31.82) 491.82(41.78)

*N* = 92, *M* Mean, *SD* Standard Deviation, *RTV* Reaction Time Variability; T scores converted from raw scores based on age and sex are presented for Teacher-rated Inattention; Raw scores are presented for Omission errors and RTV

## Results

Table 2 shows the descriptive statistics for all study variables. Bivariate correlations among all study variables are presented in Table 3. Bivariate correlation analysis showed a weak negative correlation between the NGRT and Omission errors, *r*(92) = –0.40, *p* < 0.01 and a weak negative correlation between the NGRT and Teacher-rated Inattention, *r*(92) = –0.21, *p* < 0.05. Therefore, more inattentive children as assessed by AULA were more likely to achieve poor reading scores on the NGRT. Such correlations were not found for RTV. Bivariate correlation analysis showed a weak negative correlation between RTV and MRQ, *r*(92) = –0.27, *p* < 0.05. Thus, less motivated children as shown by the MRQ were more likely to experience lapses in attention as measured by AULA. Similar correlations were not found between the Omission errors and MRQ and Teacher-rated Inattention and MRQ. Surprisingly, no correlation was found between the NGRT and MRQ. Children also achieved higher, but not significantly different Reading comprehension scores for Story A across both conditions, *M* = 9.04, *SD* = 3.94 compared with Story B, *M* = 8.42, *SD* = 3.31, *p* = 0.122 (two-tailed). Similarly, children achieved higher, but not significantly different Enjoyment scores for Story A across both conditions, *M* = 39.01, *SD* = 7.93 compared with Story B, *M* = 38.22, *SD* = 6.44, *p* = 0.319.

**Table 2:** Descriptive statistics for all variables

Measures	*N*	*M (SD*)	*Range*	*K*	*S*
Baseline reading ability NGRT	92	100.15 (11.83)	78—131	–0.13	0.52
Baseline reading motivation MRQ	92	110.53 (16.45)	71—144	–0.30	–0.28
Reading intervention Reading difference	92	0.97 (3.73)	-9—12	0.25	0.20
Enjoyment difference	92	1.38 (7.10)	-18—19	–0.19	–0.14
Conners 3 Teacher scale Teacher-rated Inattention	92	52.15 (10.57)	42—90	0.99	1.23
AULA Omissions	92	39.72 (32.73)	2—127	–0.57	0.78
RTV	92	405.43 (77.14)	237.78 -577.56	–0.52	0.20

*N* = 92, *M* Mean, *SD* Standard deviation, *K* Kurtosis, *S* Skewness, *NGRT* New Group Reading Test (standardised scores based on age), *MRQ* Motivation for Reading Questionnaire (raw scores), Conners 3 Teacher scores converted to T scores based on age and sex, *AULA*  Advanced Virtual Reality Tool for the Assessment of Attention (raw scores), Omissions  Omission errors, *RTV* Reaction Time Variability

**Table 3:** Bivariate correlations among variables

Variable	1	2	3	4	5	6	7
1. NGRT							
2. MRQ	0.08						
3. Reading difference	–0.07	0.05					
4. Enjoyment difference	0.04	0.01	0.17				
5. Teacher-rated Inattention	–0.21^*^	–0.10	0.02	–0.06			
6. Omissions	–0.40^**^	–0.11	–0.05	0.16	0.47^**^		
7. RTV	–0.08	–0.27^*^	0.01	0.12	0.30^**^	0.45^**^	

*N* = 92. Reading difference scores and Enjoyment difference scores were measured subtracting raw scores in No Choice by raw scores in Choice. Significant correlations are marked in bold

*p < 0.05; **p < 0.01

Hypothesis 1: Choice will increase the reading comprehension and enjoyment of children compared with no choice.

A repeated measures ANOVA showed that there was a two-tailed statistically significant difference, *F*(1,91) = 6.18, *p* = 0.015, ${\eta }_{p}^{2}$^4^ = 0.06. Children achieved greater Reading comprehension scores in the Choice, *M* = 9.22, *SD* = 3.68 than No Choice condition, *M* = 8.25, *SD* = 3.55. Similarly, a repeated measures ANOVA tested whether children reported greater Enjoyment in the Choice than No Choice. There was no significant difference in Enjoyment scores across the conditions, *F*(1,91) = 3.48, *p* = 0.065, ${\eta }_{p}^{2}$ = 0.04. Story and condition order affected Reading comprehension or Enjoyment scores (see Online Resource).

Hypothesis 2: Choice will increase the reading comprehension and enjoyment of children with both severe inattention and no attentional difficulties compared with no choice.

Hypothesis 3: There will be an interaction between choice and attention, such that choice will increase particularly the reading comprehension and enjoyment of severely inattentive children than with those with no attentional difficulties.

### Whole-sample Analysis

Positive correlations were not reported between difference scores for both Reading comprehension and Enjoyment and Attention (Omission errors, RTV, Teacher-rated Inattention), showing that more inattentive children did not achieve greater Reading comprehension and Enjoyment in Choice than No Choice (Table 3).

### Mixed Repeated Measures ANOVA’s for Reading Comprehension

**Fig1:**
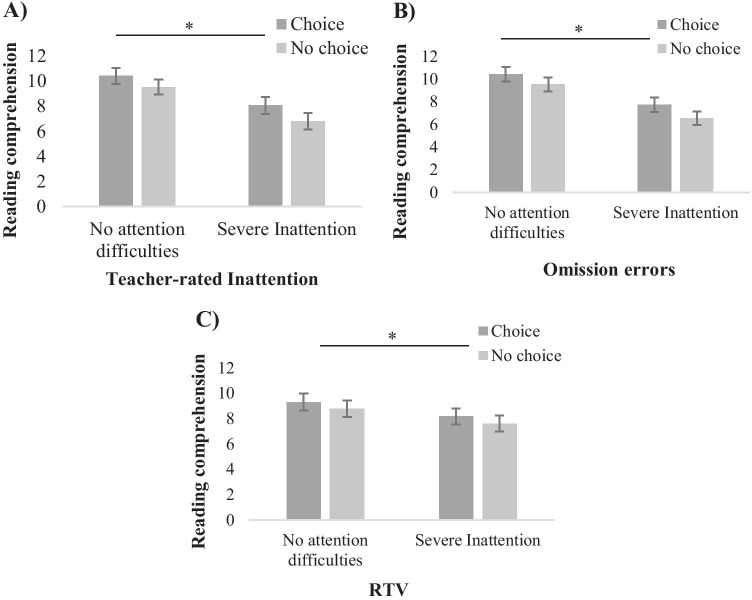
(**A**) Reading comprehension scores by Teacher-rated Inattention and Condition (**B**) Reading comprehension scores by Omission errors and Condition (**C**) Reading comprehension scores by RTV and Condition. Choice effects were significant across the sample. Error bars represent standard errors. * *p* < 0.05 (two-tailed). Asterisks and lines represent significance

A mixed 2 × 3 ANOVA was employed with Choice as the within-subjects variable and trichotomized Teacher-rated Inattention as the between-subjects variable to address *Hypotheses 2* and *3*. There was a main effect of Choice, *F*(1,89) = 6.17, *p* = 0.015, ${\eta }_{p}^{2}$ = 0.65. There was also a main effect of Teacher-rated Inattention, *F*(2,89) = 5.68, *p* = 0.005, ${\eta }_{p}^{2}$ = 0.11. Following Bonferroni adjustment for multiple comparisons, pairwise comparisons showed that children with severe inattention achieved significantly poorer Reading comprehension scores than children with no attentional difficulties as rated by teachers (*p* = 0.004); however, both groups did better in Choice (*M* = 10.42, *SD* = 3.36 for children with no attentional difficulties; *M* = 8.07, *SD* = 4.00 for children with severe inattention) compared with No Choice (*M* = 9.55, *SD* = 3.66 for children with no attentional difficulties; *M* = 6.82, *SD* = 3.20 for children with severe inattention). There was no significant interaction between Choice and Teacher-rated Inattention, *F*(2,89) = 0.12, *p* = 0.891, ${\eta }_{p}^{2}$ <0.001. A second mixed 2 × 3 ANOVA was employed with Choice as the within- subjects variable and trichotomized Omission errors as the between-subjects variable. There was a main effect of Choice, *F*(1,89) = 6.04, *p* = 0.016, ${\eta }_{p}^{2}$ = 0.06. There was a main effect of Omission errors, *F*(2,89) = 7.74, *p* = 0.001, ${\eta }_{p}^{2}$ = 0.15. Following Bonferroni adjustment for multiple comparisons, pairwise comparisons showed that children with severe inattention produced significantly poorer Reading comprehension scores than children with no attentional difficulties as indicated by Omission errors (*p* = 0.001). Taken these results together, children with severe attentional difficulties achieved lower Reading comprehension scores than children with no attentional difficulties, however, both groups had better reading comprehension in Choice (*M* = 10.43, *SD* = 3.58 for children with no attentional difficulties; *M* = 7.74, *SD* = 3.59 for children with severe inattention) compared with No Choice (*M* = 9.53, *SD* = 3.01 for children with no attentional difficulties; *M* = 6.55, *SD* = 3.43 for children with severe inattention). There was no interaction between Choice and Omission errors, *F*(2,89) = 0.89, *p* = 0.915, ${\eta }_{p}^{2}$ = 0.02. A further 2 × 3 mixed ANOVA was applied with Choice as the within-subjects variable and trichotomized RTV as the between-subjects variable. There was a main effect of Choice, *F*(1,89) = 6.14, *p* = 0.015, ${\eta }_{p}^{2}$ = 0.07 such that children achieved greater Reading comprehension in Choice (*M* = 9.30, *SD* = 3.84 for children with no attentional difficulties; *M* = 8.16, *SD* = 3.74 for children with severe inattention) than No Choice (*M* = 8.77, *SD* = 3.83 for children with no attentional difficulties; *M* = 7.61, *SD* = 3.57 for children with severe inattention). There was no main effect of RTV, *F*(2,89) = 1.83, *p* = 0.167, ${\eta }_{p}^{2}$ = 0.04. There was no interaction between Choice and RTV, *F*(2,89) = 1.19, *p* = 0.310, ${\eta }_{p}^{2}$ = 0.03 (see Fig. 1).

### Mixed Repeated-Measures ANOVA’s for Reading Enjoyment

A mixed 2 × 3 ANOVA was employed with Choice as the within-subjects variable and trichotomized Teacher-rated Inattention as the between-subjects variable to address *Hypotheses 2* and *3* (see Online Resource for Ms and SDs). There was no main effect of Choice, *F*(1,89) = 3.37, *p* = 0.070, ${\eta }_{p}^{2}$ = 0.04. There was no main effect of Teacher-rated Inattention, *F*(2,89) = 0.35, *p* = 0.703, ${\eta }_{p}^{2}$ = 0.01. There was no interaction between Choice and Teacher-rated Inattention, *F*(2,89) = 0.06, *p* = 0.941, ${\eta }_{p}^{2}$ < 0.001. A mixed ANOVA with Choice as the within-subjects variable and trichotomized Omission errors as the between-subjects variable was employed. There was no main effect of Choice, *F*(1,89) = 3.58, *p* = 0.062, ${\eta }_{p}^{2}$ = 0.04 and no main effect of Omission errors, *F*(2,89) = 1.10, *p* = 0.338, ${\eta }_{p}^{2}$ = 0.02. There was no interaction between Enjoyment scores during Choice and Omission errors, *F*(2,89) = 2.90, *p* = 0.060, ${\eta }_{p}^{2}$ = 0.06. Finally, a mixed ANOVA was employed with Choice as the within-subjects variable and trichotomized RTV as the between-subjects variable. There was no main effect of Choice, *F*(1,89) = 3.39, *p* = 0.069, ${\eta }_{p}^{2}$ = 0.04. There was no main effect of RTV, *F*(2,89) = 2.82, *p* = 0.065, ${\eta }_{p}^{2}$ = 0.06. Again, no interaction^5^ was found between Choice and RTV, *F*(2,89) = 0.41, *p* = 0.664, ${\eta }_{p}^{2}$ = 0.01.

## Discussion

We tested the hypotheses that 1) eight- to- nine year old children in mainstream schools would show increased reading comprehension and enjoyment when given a (perceived) choice of a story rather than no choice; 2) choice effects would be present for all children, irrespective of the severity of inattention (Teacher-rated Inattention, Omission errors and RTV); and 3) choice effects would be significantly stronger for children with severe inattention than those with no attentional difficulties. Children participated in a reading intervention with two conditions, a Choice where they selected one out of two stories and a No Choice where they were assigned a story. They completed a virtual reality task designed to assess inattention in a classroom setting and measures of baseline reading ability and motivation. Teachers rated children’s inattention.

Testing of *Hypothesis 1* showed that all children achieved greater reading comprehension, when they had the option to choose their story compared with no choice. These findings replicated Fridkin’s study (2018), which also showed that choice improved reading comprehension in eight- to- nine year old children, although they did not report significant choice effects on reading enjoyment. These findings are partially in line with research, which found that choice increases affective engagement (e.g. attitudes, enjoyment), but not necessarily cognitive engagement (e.g. test scores) (Flowerday & Schraw, 2003). Differences in the effects of choice on reading comprehension may be due to the operationalisation of choice. Similar to Fridkin (2018), children chose their story based on generic story-related reading materials (titles, prologues, reviews), which may have increased children’s perceptions of task meaningfulness. Children who perceive their reading as personally meaningful are more likely to attend to and invest themselves in this (Katz & Assor, 2007). By developing new stories for this study and offering perceived text choice, we controlled for story knowledge and personal (topic) interest. Therefore, these findings gain greater value as they propose that choice triggers a fleeting cognitive and affective response (situational interest) that occurs independent of personal reading interests. This offers supportive evidence for the value of choice and potentially other triggers for situational interest, valuable tools for educators who struggle to motivate those children who have not yet developed a genuine interest for reading (Hidi & Harackiewicz, 2000). It should be noted, however, that although choice may serve as a booster of situational interest with cognitive and potentially affective benefits, it is important to explore longitudinally how situational interest can evolve into more genuine forms of interest (individual interest) and result subsequently in increased motivation and improved reading.

Choice effects on reading enjoyment were not significant, although there was a non-significant trend (*p* < 1) with children achieving greater enjoyment scores in Choice than No Choice. In theoretical terms, choice has been viewed as a trigger of situational interest, which represents the first phase of interest development (Hidi & Renninger, 2006). During this phase, children may not be fully aware of their situational interest and enjoyment. Thus, they may lack the ability to report on these constructs, which provides an explanation for our failure to report significant choice effects (Renninger & Hidi, 2016). Use of self-reports with children may not necessarily be a reliable measure due to the nature of situational interest. It is important to acknowledge, however, the possibility that choice may not increase enjoyment similar to reading comprehension. Nonetheless, our findings show that choice could motivate cognitive engagement with reading, regardless of whether children are engaged on a more affective level.

In order to test *Hypotheses 2* and *3*, we applied the trichotomization method after checking for an interaction in the full sample that tested for significant correlations between reading difference scores, enjoyment difference scores and attention variables (Teacher-rated Inattention, Omission errors and RTV). Results remained consistent across all the analyses for each of the different measures of inattention. The partial confirmation of *Hypothesis 2* suggests that choice can improve the reading comprehension (not the reading enjoyment) of all children compared with no choice, including those children with attentional difficulties, a risk factor for reading difficulties (Rabiner et al., 2016). This is consistent with the mechanisms proposed by the Four-Phase Model of Interest Development (Hidi & Renninger, 2006), specifically relating to attention. Within this model, choice, among other situational factors (e.g. novelty), triggers subconscious interest resulting in an effortless allocation of attention and leading to increased reading performance. Although choice effects ranged from small-to-moderate in this study, this is nonetheless notable given the fairly minor nature of the situational manipulation. It suggests that manipulating situational factors could provide a way to support the reading of children with different attentive profiles. Whilst there are some studies with small sample sizes that demonstrate the benefits of motivation during learning for children with ADHD (Powell & Nelson, 1997), the effects of motivation in children will a less severe profile are less known and not always reported clearly in literature (Gaastra et al., 2016). Our findings around the positive choice effects on the reading outcomes of children with varying degrees of inattention have important practical implications. Inclusive approaches that target the whole classroom rather than individual children could prove more attractive and easily-applicable for general education teachers (Moore et al., 2019), who may have limited freedom over classroom instruction and planning due to high-stakes testing.

For *Hypothesis* 3, we drew upon theories (e.g. Delay aversion, Optimal stimulation) and research in ADHD (Luman et al., 2005), which posit that motivation is specifically implicated in the attention of children with ADHD, to further test for such motivational effects on the reading of children with relatively severe inattention in mainstream schools. We did not confirm *Hypothesis 3*. Choice did not have a greater effect on the reading comprehension and enjoyment of severely inattentive children than their peers with no attentional difficulties. Our findings failed to extend motivational theories in ADHD and offer empirical validation for the pronounced effects of motivation on severely inattentive children with no diagnosis.

Motivational practices were found particularly effective at improving the cognitive performance of children with diagnosed inattention and/or hyperactivity/impulsivity, compared with children with no such characteristics (Luman et al., 2005; Ma et al., 2016). A possible explanation for the lack of interaction between choice and attention in our study is that there is a fundamental difference in processing between children with non-diagnosed attentional difficulties and children diagnosed with ADHD. Previous literature in ADHD examined the positive effects of extrinsic motivators (e.g. rewards) rather than intrinsic motivators on behavior (Ma et al., 2016), which adds a further challenge when interpreting the non-significant choice effects with non-diagnosed children. Considering that situational interest, as promoted by choice, represents the first phase of personal interest development (Hidi & Renninger, 2006), it is likely that provision of text choice may not be sufficient to increase especially the reading outcomes of severely inattentive children. It is necessary to explore more extensively whether other intrinsic motivators (e.g. individual interest) could have more salient effects on the reading outcomes of inattentive children. It also remains unknown whether extrinsic motivators (e.g. reward) could have particular positive effects on the learning of children with non-diagnosed severe inattention, similar to children diagnosed with ADHD. Also, most studies in ADHD reported positive motivational effects on behavioral performance as measured via Go/No-Go tasks (Marx et al., 2018) and did not examine for such effects on school-related subjects including reading. Contrary to these behavioral tasks, reading represents a cognitively demanding activity and therefore motivational effects may differ for children with severe inattention and/or ADHD. Although the stories of our study were tested repeatedly with primary-aged children, it is probable that shorter passages with a limited set of questions are more appropriate when examining motivational effects considering that non-diagnosed inattentive children could struggle with working memory (Gray et al., 2015). Further reading interventions that examine the full spectrum of intrinsic and extrinsic motivators with both community and clinical samples could shed more light on the effects of different motivational forces on the reading outcomes of children with various attentive profiles.

Severely inattentive children as assessed by AULA were more likely to achieve poorer reading scores on the NGRT. Such correlations were not found for RTV and Teacher-rated Inattention. Surprisingly, no association was found between baseline reading ability (NGRT) and baseline reading motivation (MRQ). Similar findings were also reported in Fridkin’s study (2018). These findings might be explained by a decline of reading motivation and positive reading attitudes particularly in children aged 8 to 11 years old, partially due to developmental changes (Sainsbury & Schagen, 2004). Furthermore, although the MRQ is a reliable measure (a = 0.85) that has been used widely with children, reflection on past reading behavior may be a relatively challenging cognitive process particularly for severely inattentive children. Findings should also be examined carefully considering that the internal consistency of the stories was good and not excellent (a = 0.77 for Story A; a = 0.66 for Story B). Children achieved higher, but no significantly different reading and enjoyment scores for Story A than Story B, despite previous amendments to the story questions. A cross-over design was selected to control for the confounding effects of story type and mitigate this problem. Our study included a repeated measures design that counterbalanced the story and condition order, a multi-method assessment of attention and a diverse sample of children with different attentive profiles. Nevertheless, findings require further investigation. For example, due to our focus on the interaction between choice and attention, we did not control for socioeconomic status and intelligence, although both these factors could be associated with reading comprehension and attentional difficulties.

In conclusion, this is the first study to explore empirically and report that choice is an important motivational practice that increases the reading comprehension of children with severe inattention and those with no attentional difficulties alike. Choice did not significantly increase enjoyment, although there was a trend with children achieving greater enjoyment scores in Choice than No Choice. Choice did not increase the reading comprehension and enjoyment of the severely inattentive children independently. These findings do not fully support previous studies that found more salient effects of motivational practices (e.g. extrinsic motivators) for the behavior of children with ADHD. These novel findings extend our knowledge about the importance of autonomy-supportive practices, such as choice provision, for the reading of all children in mainstream classrooms. They also provide a starting point to further examine choice effects in young readers with varying attentive profiles (community, clinical) to establish generalizability, with the aim of informing the reading practices of educators. Considering teachers’ high workload, stress and limited freedom over classroom learning due to the exam-oriented character of school education (Ofsted, 2019), inclusive reading practices that address the educational needs of the entire classroom rather than individual children could offer effective practical tools to support general education teachers. 

## Supplementary Information

Below is the link to the electronic supplementary material.

Supplementary file1 (DOCX 48 KB)

## Data Availability

Available on UCL Data Repository.

## Footnotes

1 *Challenging behavior* causes harm to the person presenting this particular behavior or to those people around them. This term refers to self-injurious, aggressive and non-directed behaviors (e.g. stealing and property damage) (ADHD Foundation, 2018).

2 Data were collected as part of a larger study.

3 Means and Standard Deviations for Reading Comprehension by Group and Condition are provided in the Online Resource for the reader’s reference.

4 Means and Standard Deviations for Reading Comprehension by Group and Condition are provided in the Online Resource for the reader’s reference.

5 No significant interactions between Choice and Attention were found for Reading comprehension and Enjoyment in further moderation analyses (see Online Resource), confirming the results of the whole sample and trichotomization analyses.

## References

[CR1] ADHD Foundation, A. (2018). *Factsheet: Managing challenging behaviour*. https://www.adhdfoundation.org.uk/wp-content/uploads/2018/01/Factsheet-Managing-Challenging-Behaviour.pdf

[CR2] American Psychiatric Association (2013). Diagnostic and statistical manual of mental disorders: DSM-5.

[CR3] Areces D, Dockrell J, García T, González-Castro P, Rodríguez C (2018). Analysis of cognitive and attentional profiles in children with and without ADHD using an innovative virtual reality tool. PLoS One.

[CR4] Beike SM, Zentall SS (2012). The Snake Raised Its Head: Content Novelty Alters the Reading Performance of Students At Risk for Reading Disabilities and ADHD. Journal of Educational Psychology.

[CR5] Burge B, Styles B, Brzyska B, Cooper L, Shamsan Y, Saltini F, Twist L (2010). New Group Reading Test (NGRT).

[CR6] Cherkes-Julkowski M, Stolzenberg J, Hatzes N (1995). Methodological issues in assessing the relationship among ADD, medication effects and reading performance. Learning Disabilities: A Multidisciplinary Journal.

[CR7] Clark, C., & Phythian-Sence, C. (2008). *Interesting choice the (relative) importance of choice and interest in reader engagement*. London: National Literacy Trust. https://cdn.literacytrust.org.uk/media/documents/2008_07_20_free_research_-_interesting_choice_review_2008_FfPOxNX.pdf

[CR8] Cohen, J. (1988). *Statistical Power Analysis for the Behavioral Sciences* (2nd ed.). Lawrence Erlbaum Associates.

[CR9] Conners, C. K. (2008). *Conners 3rd edition manual*. Toronto, Ontario, Canada: Multi-Health Systems.

[CR10] Department for Education (DfE). (2012). *Research evidence on reading for pleasure: Education standards research team*. https://assets.publishing.service.gov.uk/government/uploads/system/uploads/attachment_data/file/284286/reading_for_pleasure.pdf

[CR11] Díaz-Orueta U, Garcia-López C, Crespo- Eguílaz N, Sánchez-Carpintero Ro, Climent G, Narbona J (2013). AULA virtual reality test as an attention measure: Convergent validity with Conners Continuous Performance Test. Child Neuropsychology.

[CR12] Dunlap G, DePerczel M, Clarke S, Wilson D, Wright S, White R, Gomez A (1994). Choice making to promote adaptive behaviour for students with emotional and behavioral challenges. Journal of Applied Behavior Analysis.

[CR13] Dupaul GJ, Eckert TL, Vilardo B (2012). The Effects of School-Based Interventions for Attention Deficit Hyperactivity Disorder: A Meta-Analysis 1996–2010. School Psychology Review.

[CR14] Faul F, Erdfelder E, Lang A-G, Buchner A (2007). GPower 3: A flexible statistical power analysis program for the social, behavioral, and biomedical sciences. Behavior Research Methods.

[CR15] Flowerday T, Schraw G, Stevens J (2004). The Role of Choice and Interest in Reader Engagement. The Journal of Experimental Education.

[CR16] Flowerday T, Schraw G (2003). Effect of Choice on Cognitive and Affective Engagement. The Journal of Educational Research (Washington, D.c.).

[CR17] Fridkin, L. (2018). *The impact of motivation on children's reading comprehension: differential effects of gender and ability* [Doctoral dissertation, UCL Institute of Education]. UCL Database.

[CR18] Gaastra GF, Groen Y, Tucha L, Tucha O (2016). The effects of classroom interventions on off-task and disruptive classroom behavior in children with symptoms of attention-deficit/hyperactivity disorder: a meta-analytic review. PLoS One.

[CR19] Gathercole SE, Astle DA, Manly T, Holmes J, the CALM Team (2018). Cognition and behaviour in learning difficulties and ADHD: A dimensional approach. BioRxiv.

[CR20] Ghelani K, Sidhu R, Jain U, Tannock R (2004). Reading comprehension and reading related abilities in adolescents with reading disabilities and attention-deficit/hyperactivity disorder. Dyslexia.

[CR21] GL Assessment. (2018). *Technical Information: New Group Reading Test ® (NGRT) Digital Edition*. https://www.gl-assessment.co.uk/media/294737/ngrt-technical-final-proof.pdf

[CR22] Gray SA, Rogers M, Martinussen R, Tannock R (2015). Longitudinal relations among inattention, working memory, and academic achievement: Testing mediation and the moderating role of gender. PeerJ.

[CR23] Guthrie JT, Hoa ALW, Wigfield A, Tonks SM, Humenick NM, Littles E (2007). Reading motivation and reading comprehension growth in the later elementary years. Contemporary Educational Psychology.

[CR24] Hagmar L, Brøgger A, Hansteen IL, Heim S, Högstedt B, Knudsen L, Lambert B, Linnainmaa K, Mitelman F, Nordenson I (1994). Cancer risk in humans predicted by increased levels of chromosomal aberrations in lymphocytes: Nordic study group on the health risk of chromosome damage. Cancer Research (Chicago, Ill.).

[CR25] Hidi S, Renninger KA (2006). The Four-Phase Model of Interest Development. Educational Psychologist.

[CR26] Hidi S, Harackiewicz JM (2000). Motivating the academically unmotivated: A critical issue for the 21st century. Review of Educational Research.

[CR27] Hurry J, Flouri E, Sylva K (2018). Literacy Difficulties and Emotional and Behavior Disorders: Causes and Consequences. Journal of Education for Students Placed at Risk (JESPAR).

[CR28] Iriarte Y, Diaz-Orueta U, Cueto E, Irazustabarrena P, Banterla F, Climent G (2016). AULA-Advanced Virtual Reality Tool for the Assessment of Attention: Normative Study in Spain. Journal of Attention Disorders.

[CR29] Izzo VA, Donati MA, Novello F, Maschietto D, Primi C (2019). The Conners 3-short forms: Evaluating the adequacy of brief versions to assess ADHD symptoms and related problems. Clinical Child Psychology and Psychiatry.

[CR30] Katz I, Assor A (2006). When Choice Motivates and When It Does Not. Educational Psychology Review.

[CR31] Kendeou P, van den Broek P, Helder A, Karlsson J (2014). A Cognitive View of Reading Comprehension: Implications for Reading Difficulties. Learning Disabilities Research and Practice.

[CR32] Kieffer MJ, Vukovic RK, Berry D (2013). Roles of attention shifting and inhibitory control in fourth-grade reading comprehension. Reading Research Quarterly.

[CR33] Koo TK, Li MY (2016). A Guideline of Selecting and Reporting Intraclass Correlation Coefficients for Reliability Research. Journal of Chiropractic Medicine.

[CR34] Lahey BB, Willcutt EG (2010). Predictive Validity of a Continuous Alternative to Nominal Subtypes of Attention-Deficit/Hyperactivity Disorder for DSM-V. Journal of Clinical Child and Adolescent Psychology.

[CR35] Luman M, Oosterlaan J, Sergeant J (2005). The impact of reinforcement contingencies on AD/HD: A review and theoretical appraisal. Clinical Psychology Review.

[CR36] Lumley T, Diehr P, Emerson S, Chen L (2002). The Importance of the normality assumption in large public health data sets. Annual Review of Public Health.

[CR37] Ma I, van Holstein M, Mies GW, Mennes M, Buitelaar J, Cools R, Cillessen AHN, Krebs RM, Scheres A (2016). Ventral striatal hyperconnectivity during rewarded interference control in adolescents with ADHD. Cortex.

[CR38] Marx, I., Hacker, T., Yu, X., Cortese, S., & Sonuga-Barke, E. (2018). ADHD and the choice of small immediate over larger delayed rewards: a comparative meta-analysis of performance on simple choice-delay and temporal discounting paradigms. *Journal of Attention Disorders* 1–17. 10.1177/1087054718772138 10.1177/1087054718772138 29806533

[CR39] Merrell C, Sayal K, Tymms P, Kasim A (2017). A longitudinal study of the association between inattention, hyperactivity and impulsivity and children's academic attainment at age 11. Learning and Individual Differences.

[CR40] Miller AC, Fuchs D, Fuchs LS, Compton D, Kearns D, Zhang W, Yen L, Patton S, Kirchner DP (2014). Behavioral attention: A longitudinal study of whether and how It influences the development of word reading and reading comprehension among at-risk readers. Journal of Research on Educational Effectiveness.

[CR41] Miller AC, Keenan JM, Betjemann RS, Willcutt EG, Pennington BF, Olson RK (2013). Reading comprehension in children with ADHD: Cognitive underpinnings of the centrality deficit. Journal of Abnormal Child Psychology.

[CR42] Moore DA, Richardson M, Gwernan-Jones R, Thompson-Coon J, Stein K, Rogers M, Garside R, Logan S, Ford TJ (2019). Non-Pharmacological Interventions for ADHD in School Settings: An Overarching Synthesis of Systematic Reviews. Journal of Attention Disorders.

[CR43] Ofsted. (2019). *Teacher well-being at work in schools and further education providers.* https://assets.publishing.service.gov.uk/government/uploads/system/uploads/attachment_data/file/819314/Teacher_well-being_report_110719F.pdf

[CR44] Patall EA (2013). Constructing motivation through choice, interest, and interestingness. Journal of Educational Psychology.

[CR45] Powell S, Nelson B (1997). Effects of choosing academic assignments on a student with Attention Deficit Hyperactivity Disorder. Journal of Applied Behavior Analysis.

[CR46] Proctor C, Linley PA, Maltby J (2010). Very Happy Youths: Benefits of Very High Life Satisfaction Among Adolescents. Social Indicators Research.

[CR47] Purvis KL, Tannock R (2000). Phonological Processing, Not Inhibitory Control, Differentiates ADHD and Reading Disability. Journal of the American Academy of Child and Adolescent Psychiatry.

[CR48] Rabiner DL, Carrig MM, Dodge KA (2016). Attention Problems and Academic Achievement: Do Persistent and Earlier-Emerging Problems Have More Adverse Long-Term Effects?. Journal of Attention Disorders.

[CR49] Rabiner D, Coie JD (2000). Early Attention Problems and Children's Reading Achievement: A Longitudinal Investigation. Journal of the American Academy of Child and Adolescent Psychiatry.

[CR50] Renninger KA, Hidi S (2016). The power of interest for motivation and engagement.

[CR51] Rogers M, Tannock R (2018). Are Classrooms Meeting the Basic Psychological Needs of Children With ADHD Symptoms? A Self-Determination Theory Perspective. Journal of Attention Disorders.

[CR52] Russell G, Rodgers LR, Ukoumunne OC, Ford T (2013). Prevalence of Parent-Reported ASD and ADHD in the UK: Findings from the Millennium Cohort Study. Journal of Autism and Developmental Disorders.

[CR53] Ryan RM, Deci EL (2000). Self-determination theory and the facilitation of intrinsic motivation, social development, and well-being. American Psychologist.

[CR54] Sainsbury M, Schagen I (2004). Attitudes to reading at ages nine and eleven. Journal of Research in Reading.

[CR55] Scheffer J (2002). Dealing with missing data. Research Letters in the Information and Mathematical Sciences.

[CR56] Sexton CC, Gelhorn HL, Bell JA, Classi PM (2012). The Co-occurrence of Reading Disorder and ADHD: Epidemiology, Treatment, Psychosocial Impact, and Economic Burden. Journal of Learning Disabilities.

[CR57] Shogren KA, Faggella-Luby MN, Sung Jik B, Wehmeyer ML (2004). The Effect of Choice-Making as an Intervention for Problem Behavior: A Meta-Analysis. Journal of Positive Behavior Interventions.

[CR58] Sierens E, Vansteenkiste M, Goossens L, Soenens B, Dochy F (2009). The synergistic relationship of perceived autonomy support and structure in the prediction of self-regulated learning. British Journal of Educational Psychology.

[CR59] Sims DM, Lonigan CJ (2013). Inattention, Hyperactivity, and Emergent Literacy: Different Facets of Inattention Relate Uniquely to Preschoolers' Reading-Related Skills. Journal of Clinical Child & Adolescent Psychology.

[CR60] Smith ZR, Langberg JM, Cusick CN, Green CD, Becker SP (2019). Academic motivation deficits in adolescents with ADHD and associations with academic functioning. Journal of Abnormal Child Psychology.

[CR61] Smith ZR, Langberg JM (2018). Review of the evidence for motivation deficits in youth with ADHD and their association with functional outcomes. Clinical Child and Family Psychology Review.

[CR62] Sonuga-Barke EJS (2005). Causal models of attention-deficit/hyperactivity disorder from: Common simple deficits to multiple developmental pathways. Biological Psychiatry.

[CR63] Su Y-L, Reeve J (2011). A meta-analysis of the effectiveness of intervention programs designed to support autonomy. Educational Psychology Review.

[CR64] Tamm L, Epstein JN, Denton CA, Vaughn AJ, Peugh J, Willcutt EG (2014). Reaction time variability associated with reading skills in poor readers with ADHD. Journal of the International Neuropsychological Society.

[CR65] The MTA Cooperative Group (2004). National Institute of Mental Health Multimodal Treatment Study of ADHD Follow-up: 24-Month Outcomes of Treatment Strategies for Attention-Deficit/Hyperactivity Disorder. Pediatrics (Evanston).

[CR66] Volkow ND, Wang GJ, Newcorn JH, Kollins SH, Wigal TL, Telang F, Fowler JS, Goldstein RZ, Klein N, Logan J, Wong C, Swanson JM (2011). Motivation deficit in ADHD is associated with dysfunction of the dopamine reward pathway. Molecular Psychiatry.

[CR67] Wigfield A, Gladstone JR, Turci L (2016). Beyond Cognition: Reading Motivation and Reading Comprehension. Child Development Perspectives.

[CR68] Wigfield A, Guthrie JT (1997). Relations of Children's Motivation for Reading to the Amount and Breadth of Their Reading. Journal of Educational Psychology.

[CR69] Willcutt EG (2012). The Prevalence of DSM-IV Attention-Deficit/Hyperactivity Disorder: A Meta-Analytic Review. Neurotherapeutics.

[CR70] Willcutt EG, Betjemann RS, McGrath LM, Chhabildas NA, Olson RK, DeFries JC, Pennington BF (2010). Etiology and neuropsychology of comorbidity between RD and ADHD: The case for multiple-deficit models. Cortex.

[CR71] Zentall S (1975). Optimal Stimulation as Theoretical Basis of Hyperactivity. American Journal of Orthopsychiatry.

