# Supplementary material for: The Effects of Choice on the Reading Comprehension and Enjoyment of Children with Severe Inattention and no Attentional Difficulties
Source: Res Child Adolesc Psychopathol. 2021 Jun 21;49(11):1403–17. doi: 10.1007/s10802-021-00835-8 (PMC8455393; doi:10.1007/s10802-021-00835-8)
Supplement: Supplementary file 1 — Supplementary file1 (DOCX 48 KB) [file 10802_2021_835_MOESM1_ESM.docx]

**Electronic Supplementary Material**

**The effects of choice on the reading comprehension and enjoyment of children with severe inattention and no attentional difficulties**

**Choice order and story effects on reading comprehension and enjoyment**

A mixed ANOVA with Choice as the within-subjects variable and Choice order (First, Second) as the between-subjects variable was conducted to test for any effects of presentation of choice on reading comprehension scores. There was a main effect of Choice *F*(1,90) = 6.38, *p* = .013,$\eta_{p}^{2}$ = 0.07. There was no main effect of Choice order, *F*(1,90) = 0.04, *p* = .840, $\eta_{p}^{2}$< .001. There was no interaction between Reading scores across both conditions and Choice order *F*(1,90) = 1.89, *p* = .173, $\eta_{p}^{2}$ = 0.02.

A further mixed ANOVA with Choice as the within-subjects variable and Story type (Story A in Choice condition, Story B in Choice condition) was applied to explore any effects of story type on reading comprehension scores. There was a main effect of Choice *F*(1,90) = 8.39, *p* = .005, $\eta_{p}^{2}$ = 0.09. There was no main effect of Story order *F*(1,90) < .001, *p* = .979, $\eta_{p}^{2}$ < .001. There was an interaction effect *F*(1,90) = 6.02, *p* = .016, $\eta_{p}^{2}$= 0.06, such that children’s reading comprehension scores were more positively affected by Choice when reading Story A than when reading Story B.

**FigS1** Choice condition story effects on reading comprehension scores by condition. Choice condition = Experimental, No Choice condition = Control. Error bars represent standard errors.

In terms of reading enjoyment, a mixed ANOVA with Choice as the within-subjects variable and Choice order (First, Second) as the between-subjects variable was conducted to test for any effects of presentation of choice on reading enjoyment scores. There was no main effect of Choice *F*(1,90) = 3.46, *p* = .066, $\eta_{p}^{2}$= 0.04. There was no main effect of Choice order *F*(1,90) < .001, *p* = .977, $\eta_{p}^{2}$< .001. There was no interaction between Reading enjoyment scores across both conditions and Choice order *F*(1,90) = 0.07, *p* = .796, $\eta_{p}^{2}$ < .001.

A further mixed ANOVA with Choice as the within-subjects variable and Story type (Story A in Choice condition, Story B in Choice condition) was applied to test for any effects of story type on reading enjoyment scores. There was a main effect of Choice *F*(1,90) = 4.25, *p* = .042, $\eta_{p}^{2}$ = 0.05. There was no main effect of Story order *F*(1,90) = 0.45, *p* = .503, $\eta_{p}^{2}$ = 0.01. There was no interaction effect *F*(1,90) = 1.90, *p* = .171, $\eta_{p}^{2}$ = 0.02.

**Means and Standard Deviations for Reading Comprehension and Enjoyment by Group and Condition**

**Table S1**

*Means and Standard Deviations for Reading Comprehension by Group and Condition*

| Measure | Group | *N* | *M (SD)*  choice | *M (SD)*  no choice |
| --- | --- | --- | --- | --- |
| **Conners 3**  Teacher-rated Inattention | No Inattention  Some Inattention  Severe Inattention | 33  31  28 | 10.42(3.36)  8.97(3.43)  8.07(4) | 9.53(3.01)  8.71(3.59)  6.55(3.43) |
| **AULA**  Omission errors | No Inattention  Some Inattention  Severe Inattention | 30  31  31 | 10.43(3.58)  9.52(3.47)  7.74(3.59) | 9.52(2.97)  8.45(3.62)  6.58(3.36) |
| **AULA**  RTV | No Inattention  Some Inattention  Severe Inattention | 30  31  31 | 9.30(3.84)  10.19(3.27)  8.16(3.74) | 8.77(3.83)  8.39(3.27)  7.61(3.57) |

*Note.* *N* = 92. *M* = Mean; *SD* = Standard Deviation; RTV = Reaction Time Variability; T scores converted from raw scores based on age and sex are presented for Teacher-rated Inattention; Raw scores are presented for Omission errors and RTV.

**Table S2**

| Measure | Group | *N* | *M (SD)*  choice | *M (SD)*  no choice |
| --- | --- | --- | --- | --- |
| **Conners 3**  Teacher-rated Inattention | No Inattention  Some Inattention  Severe Inattention | 33  31  28 | 39.79(7.16)  39.61(6.59)  38.40(7.96) | 38.55(5.83)  37.87(8.31)  37.25(7.63) |
| **AULA**  Omission errors | No Inattention  Some Inattention  Severe Inattention | 30  31  31 | 39.17(7.03)  39.52(7.17)  39.23(7.54) | 38.53(7.61)  39.81(6.59)  35.45(6.98) |
| **AULA**  RTV | No Inattention  Some Inattention  Severe Inattention | 30  31  31 | 39.87(6.69)  40.48(6.49)  37.58(8.11) | 39.33(8.04)  39.10(6.51)  35.39(6.60) |

*Means and Standard Deviations for Reading Enjoyment by Group and Condition*

*Note.* *N* = 92. *M* = Mean; *SD* = Standard Deviation; RTV = Reaction Time Variability; T scores converted from raw scores based on age and sex are presented for Teacher-rated Inattention; Raw scores are presented for Omission errors and RTV.

**Moderation analyses to check for interactions between Choice and Attention**

We decided to use the MEMORE macro for SPSS (available at <https://www.akmontoya.com/>) to provide further support for the results of the trichotomisation method analysis (see Montoya, 2019 for a detailed description of the MEMORE tool). Three moderation analyses for reading comprehension and enjoyment each were conducted with Choice as the within-subjects variable and each Attention variable (Teacher-rated Inattention, Omission errors and RTV) as the between-subjects (moderator) variable with 5000 samples and percentile bootstrapped 95% confidence intervals. In line with the whole-sample analysis and the trichotomisation method, no significant interactions were reported between Choice and the three Attention variables for reading comprehension and enjoyment.

**Table S3**

*Results from moderation analyses testing the interaction between Choice (reading comprehension scores) and Attention variables using the MEMORE macro in SPSS.*

| Variable | Reading comprehension scores (Choice) | | | |
| --- | --- | --- | --- | --- |
|  | *R^2^* | *MSE* | *F* | *p* |
| **Conners 3**  Teacher-rated Inattention  **AULA** | 0.0 | 14.10 | 0.02 | .884 |
| Omissions  **AULA** | 0.0 | 14.06 | 0.26 | .61 |
| RTV | 0.0 | 14.1 | 0.0 | .933 |

*N* = 92. RTV = Reaction Time Variability; T scores converted from raw scores based on age and sex are presented for Teacher-rated Inattention; Raw scores are presented for Omission errors and RTV.

**Table S4**

*Results from moderation analyses testing the interaction between Choice (reading enjoyment scores) and Attention variables using the MEMORE macro in SPSS.*

| Variable | Reading enjoyment scores (Choice) | | | |
| --- | --- | --- | --- | --- |
|  | *R^2^* | *MSE* | *F* | *p* |
| **Conners 3**  Teacher-rated Inattention  **AULA** | 0.0 | 50.78 | 0.3 | .587 |
| Omissions  **AULA** | 0.03 | 49.67 | 2.33 | .13 |
| RTV | 0.02 | 50.19 | 1.37 | .246 |

*N* = 92. RTV = Reaction Time Variability; T scores converted from raw scores based on age and sex are presented for Teacher-rated Inattention; Raw scores are presented for Omission errors and RTV.

**References**

Montoya, A. K., & Montoya, A. K. (2019). Moderation analysis in two-instance repeated measures designs: Probing methods and multiple moderator models. *Behavior Research Methods, 51*(1), 61-82. doi:10.3758/s13428-018-1088-6
